# Patients’ preferences: a discrete-choice experiment for treatment of non-small-cell lung cancer

**DOI:** 10.1007/s10198-014-0622-4

**Published:** 2014-08-19

**Authors:** Axel C. Mühlbacher, Susanne Bethge

**Affiliations:** 1 IGM Institut Gesundheitsökonomie und Medizinmanagement, Hochschule Neubrandenburg, Brodaer Straße 2, 17033 Neubrandenburg, Germany; 2 Gesellschaft für Empirische Beratung GmbH (GEB), Freiburg, Germany

**Keywords:** Discrete-choice experiment, Non-small-cell lung cancer, Patient preferences, Patient-relevant treatment attributes, I10, I15, D700

## Abstract

**Objective:**

Lung cancer is a major cause of cancer-related deaths and thus represents a global health problem. According to World Health Organization (WHO) estimates, approximately 1.37 million people die each year from lung cancer. Different therapeutic approaches as well as several treatment options exist. To date decisions on which therapies to use have largely been made by clinical experts. Comparative preference studies show that underlying weighting of treatment goals by experts is not necessarily congruent with the preferences of affected patients.

**Aim and methods:**

The aim of this empirical study was to ascertain patient preferences in relation to treatment of non-small-cell lung cancer (NSCLC). After identification of patient-relevant treatment attributes via literature review and qualitative interviews(ten) a discrete-choice experiment including seven patient-relevant attributes was conducted using a fractional factorial NGene-design. Statistical data analysis was performed using latent class models.

**Results:**

The qualitative part of this study identified outcome measures related to efficacy, side effects and mode of administration. A total of 211 NSCLC patients *(N* = 211) participated in the computer-assisted personal interview. A clear preference for an increase in “progression-free survival” (coef.: 1.087) and a reduction of “tumor-associated symptoms”(cough, shortness of breath and pain); coef.: 1.090) was demonstrated, followed by the reduction of side effects: “nausea and vomiting” (coef.: 0.605); “rash” (coef.: 0.432); “diarrhea” (coef.: 0.427); and, “tiredness and fatigue” (coef.: 0.423). The “mode of administration” was less important for participants (coef.: 0.141).

**Conclusion:**

Preference measurement showed “progression-free survival” and “tumor-associated symptoms” had a significant influence on the treatment decision. Subgroup analysis revealed that the importance of “progression-free survival” increases with increased therapy experience. Based on the presented results therapies can be designed, assessed and chosen on the basis of patient-oriented findings. As such, more effective and efficient care of patients can be achieved and benefits increased.

## Introduction: non-small-cell lung cancer

### Decision problem: evaluation of different NSCLC therapy options

With the request for greater involvement and participation on the part of patients and the general societal shift toward greater self-responsibility, self-determination and autonomy the role of the patient is changing [1]. Objective and clinically-characterized needs assessments and asssociated decisions should be supplemented by patient perspectives.

The question of optimal treatment for non-small-cell lung cancer (NSCLC) is currently being examined primarily from the experts’ perspective. Decisions about appraisal, utility and ultimately compensation of health technologies are made on the basis of these expert judgments grounded on evidence of clinical efficacy obtained in (randomized) clinical trials. The question of which decision criteria are important from the perspective of NSCLC patients and how they are weighted against each other has not been taken into consideration to date. As different preference studies have shown, the judgments of experts do not correlate with the subjective preferences of patient in some cases [2, 3]. The patients’ criteria are not necessarily congruent with the endpoints set by experts [4, 5]. In addition, each endpoint should not necessarily be considered to carry the same weight in decision-making. Finally, subgroup differences in terms of preferences between different patient populations can also occur [6].

### Research question: identification and weighting of the decision criteria

As hardly any data are available to date about patient preferences regarding treatment of NSCLC in Germany, identification and weighting of patient-relevant outcomes for decision-making is necessary. Consideration of patient preferences should improve decisions about utilization of health technologies in the short term (preference based treatment alternatives lead to higher compliance and adherence), strengthen patient orientation in the medium term (patient-centered treatment alternatives and communication enhance patient understanding) and improve clinical effects in terms of morbidity and mortality in the long term (higher compliance and adherence will lead to better treatment outcomes). Information on patient preferences should be available to all stakeholders.

Against this background the following questions are to be answered in context of an empirical study with German participants:

- Identification: What are the key decision criteria for the selection of an optimal NSCLC therapy from the patients’ perspective?
- Weighting: How do patients weight these different decision criteria when selecting the optimal NSCLC therapy?
- Subgroup: Are there differences in weighting of the relevant decision criteria for different groups of patients?

We hypothesized that the prolongation of “progression-free survival” is of highest value for patients. In addition, we wanted to test if the mode of administration contributed to overall patient benefit (was significant in the decision model). Discrete-choice experiments (DCEs) are a commonly used method of revealing patient preferences proposed by the German Health Technology Assessment (HTA) Agency [Institute for Quality and Efficiency in Health Care (IQWiG)] [7]. Therefore, this method was seen as appropriate for answering the research question.

### Objective: scientific evidence of patients’ perspectives

The aim of this empirical study was to document patient preference regarding drug treatment of NSCLC. A DCE was used to rank patient-relevant treatment characteristics at the end of the investigation. In addition, results of this survey can provide a basis for a patient-oriented evaluation of treatment options for NSCLC and assess the patients' perspective on the basis of scientific evidence. Against the backdrop of increasing integration of patients in treatment decisions insight into patient preferences gained from this analysis could also be used in the context of shared decision-making (to help professionals better understand the patients’ perspective) and could also be taken into account in (participative) decision-making about future treatment strategies [8].

Analysis of patient preference will enable patient benefit to be analysed together with, and in addition to, clinical effectiveness. Thus, preference data can create a new source of information (evidence). In this way the added value of innovative treatment options can be complemented by the patient-perceived benefit based on clinical trial data [9]. In summary, the objective of the study is to expand scientific knowledge in the field of NSCLC treatment by adding the patients’ perspective in the form of preference data.

## Decision-making context: burden of disease and treatment alternatives

### Burden of disease: prevalence and incidence

Lung cancer is a major cause of cancer-related deaths and thus represents a global health problem. Lung cancer is the most common form of cancer that occurs at an above-average frequency, especially in the developed countries of America and Europe. National geographic differences also exist with regard to prevalence. In larger cities and industrial regions the probability of developing NSCLC is usually higher [10].

According to WHO estimates, approx. 1.37 million people die each year from lung cancer [11]. With over 43,000 deaths, malignant neoplasm of the bronchus and lung (ICD-10 Pos. Nr. C-34) is the fourth leading cause of death in Germany [12]. The 5-year survival rate of patients with lung carcinoma in North America and the European countries ranges between 5.5 and 15.7 % [10]. Lung cancer is phenotypically divided into non-small-cell lung (NSCLC) and small-cell lung carcinoma (SCLC). 80–85 % of lung cancer patients suffer from NSCLC [13].

Smoking has been identified as the main risk factor for lung cancer, with the duration of smoking being the most important factor [14]. Passive smoking also increases the risk. Studies have shown that there are biological differences in the lung cancer of smokers and non-smokers [15]. Thus, patients who have what are known as adenocarcinomas are predominantly non-smokers [16]. Moreover, epidermal growth factor receptor (EGFR) mutations are more common in tumors of non-smokers [17, 18]. The main symptoms of NSCLC are cough, shortness of breath, weight loss and chest pain. In addition, patients with NSCLC appear to be more likely to suffer from chronic obstructive pulmonary disease (COPD) [19].

### Treatment (alternatives)

Treatment of NSCLC aims to delay progression of the tumor cells as long as possible, to increase survival time as much as possible, to reduce tumor-related symptoms and ultimately to maintain the patients’ quality of life or even improve it [10, 20–22]. The best treatment decision is always one that involves weighing expected benefits against possible side effects [23–27]. Potential treatment options are influenced by the stage of disease and factors such as socio-demographic status, co-morbidities and cardiopulmonary function [19, 28].

Treatment of NSCLC is based on tumor staging [Classification of Malignant Tumours (TNM classification)] and the current condition of the patient. In stages IA to IIB the primary objective is generally surgical tumor resection with a curative aim. In advanced stages chemotherapy and targeted therapies (e.g., tyrosine kinase inhibitors) for patients with certain mutations and radiotherapy are applied [22, 29, 30].

Owing to the severity of the disease in stage IIIB and IV and the associated limited average remaining life expectancy of 8–12 months fast and reliable access to care (including rehabilitation) should be ensured in addition to ongoing medical treatment. Current treatment guidelines recommend first-line cisplatin combination chemotherapy or tyrosine kinase inhibitors for prolongation of progression-free survival times, disease control and improvement in quality of life. Treatments used have to be determined individually depending on the patient’s general condition, age and any existing comorbidities. Careful monitoring of quality of life and the success of therapy has to be ensured during treatment [20, 21]. Continuing therapy (e.g., second-line chemotherapy, surgical removal of the tumor or metastasis, radiotherapy) are to be determined depending on the response to first-line therapy, as well as on the general condition of the patient and the degree of metastasis [10].

## Methods and study design: elicitation of patient preferences

### Discrete-choice experiments

The Discrete-Choice Method is a choice-based version of a conjoint analysis that was made possible by the theoretical work of Lancaster [31] and McFadden [32]. Instead of ranking or rating different therapeutic features, as is done in traditional importance elicitation formats and conjoint analyses, DCEs perform a pairwise comparison of hypothetical alternatives (i.e., differently configured therapy options) and ask the participants to choose between them [33].

Thus respondents are forced to make trade-offs between attributes and their levels. The method offers practical advantages such as closeness to reality as trade-off decisions are part of everyday life. Implementation of pairwise comparisons considerably reduced the degree of complexity of the tasks for the participants [34–36]. Therefore, DCEs are increasingly used in health economics and health service research [37, 38].

Multiple steps are involved in the structure and design of a DCE and its evaluation. Several checklists are available and were considered during the design of the study [9, 34, 36, 39].

### Study design

#### Decision model:

##### Attributes and levels

At the beginning literature research on the indication of NSCLC was conducted (PubMed, Medline, and Cochrane Library) to document the available state-of-the-art treatment options. Additionally, the method of concentric circles was used to complement the relevant literature [40]. The aim of the search was to identify potential properties and characteristics of NSCLC treatments in general and from the patients’ perspective.

Prior to the main survey a preliminary qualitative study was conducted. In *N* = 10, qualitative semi-structured interviews with NSCLC patients (*N* = 6), care givers (*N* = 3), and other cancer patients (*N* = 1) were conducted and the treatment characteristics extracted from the literature were tested. Previously identified therapeutic features were confirmed and their relevance to the patients was assessed. Furthermore, patients could name additional as yet unidentified treatment attributes that would be patient-relevant. This allowed the subjective views of the patients surveyed to be reflected. Moreover, the interviews were used to evaluate the clarity of the questionnaire, the quality of the scales used and the understandability of the attributes, levels and trade-offs included. In parallel, the quality of the instrument was optimized [41].

**Fig. 1:**
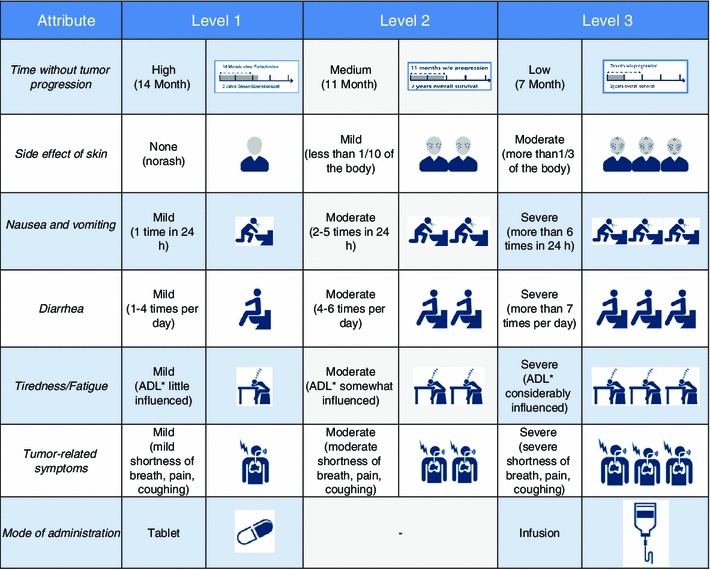
Overview of the decision model with visualizations. *ADL* activities of daily living

The final framework of the attributes used, the characteristics with explanations and associated icons are displayed in the following graph (Fig. 1). All level characteristics were based on realistic studies and confirmed by quotations from randomized controlled trials. These levels were used in the main study to create an optimal experimental design and the patient-friendly introductory texts.

As part of the literature review and interviews the “realistic range of levels” was confirmed so that this analogy could be used to establish the DCE. Description of the attribute levels was created in the style of the classification scheme by the US Department of Health and Human Services and the Office of Human Research Protection of the FDA [42]. Furthermore, a detailed patient-friendly introduction to each single attribute and related level was included in the survey. The attribute "progression-free survival" in particular was introduced to avoid any free interpretations. The participants were informed that neither overall survival nor quality of life is affected by progression-free survival within the study context.

##### Data collection plan, sample size, stratification and recruitment

The survey was conducted from May to June 2013 with computer-assisted personal interviews. Recruitment of patients for the survey was performed by an external research institute. Patients diagnosed with NSCLC stage IV who had been treated within the last 2 years and who were older than 18 years were included in the survey. Patients who did not meet all the criteria were excluded as ineligible (disqualified) as were all patients who did not complete the questionnaire (incomplete).

##### Ethical considerations

The study was a social science survey and did not contain personal data (completely anonymous survey), information on surgeries (tests, experiments, and medication), biomedical research or additional data, as is the case in many epidemiological investigations. Therefore, an ethics committee vote was not necessary. All respondents were informed about the study and its potential risks and benefits prior to participation. All respondents signed an informed consent form. They participated voluntarily and could end their participation at any time. All documents used in the study went through an internal approval process by the sponsor of the study.

#### Data collection: instrument, elicitation technique, tasks and experimental design

The final decision model was established based on the qualitative pilot study. Seven patient-relevant characteristics each described by two or three levels were extracted and included in the main survey (Fig. 1). Overall, the final questionnaire consisted of five sections. The first part included socio-demographic questions, such as age, gender, weight, height, marital status, occupation and education. This socio-demographic data may be used for subgroup analyses. The second part of the questionnaire included questions about current state of health and previous treatment. The third part included information about the attributes of the NSCLC drug therapy and realistic representations of their levels. The fourth section contained a constant sum question used for personal assessment of the treatment.

**Fig. 2:**
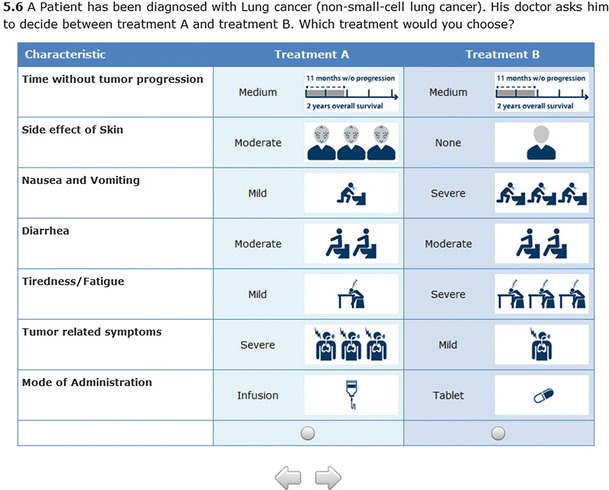
Example choice set of the discrete-choice experiment

The fifth part contained the 12 choice sets of the DCE. The subjects indicated which therapy alternative of the two presented they would choose in each case (trade-off relationships) (Fig. 2). No opt-out possibility was given. To simplify the choice for the participants each level of each attribute was displayed together with a visual aid.

**Fig. 3:**
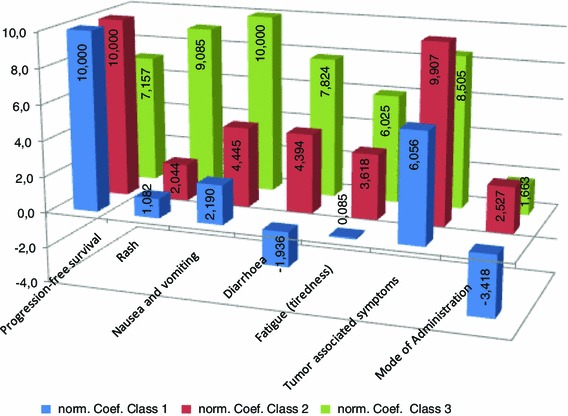
Evaluation of preference patterns for *Class 1, 2* and *3* on the basis of normalized parameters. To increase comparability of the weighting coefficients, normalization on a 10-point scale was used

##### Experimental designs

To construct the DCE choice sets an experimental design (6 × 3; 1 × 2 Design) was created using NGene software. The selected design consisted of 24 choice sets that were divided into two blocks of 12 choices so that each patient only had to make 12 decisions. The design resulted in an efficiency of D-error = 0.072242 and an A-error of 0.078126. With respect to severity of the illness and the reduced concentration capabilities of stage 4 NSCLC patients it was necessary to ease the choice tasks as much as possible. To simplify the choices the moderate types were modelled so that they were systematically overlapping, as overlap provides a way to simplify choice questions by reducing the number of attribute differences respondents have to evaluate [43]. The experimental design was therefore robust against potential recruitment problems and was easier to understand for seriously ill participants in the survey. Owing to the underlying dichotomous design, a subgroup analysis was possible when the planned sample size had been achieved.

According to the Orme calculation, the minimum sample size was *N* = 126 participants. As this calculation is currently debated in terms of being the lowest limit and given the severity of the disease, the restrictive access criteria, and the limited recruitment options, a sample size of *N* = 200 subjects was envisaged to guarantee a statistically robust estimate [44].

### Data analysis: data structure, statistical model, subgroup analysis, and interpretation

To estimate the relative importance of each treatment attribute latent class models were used with discrete or continuous random vectors and coefficients calculated using GLLAMM 2.3.20 in Stata 11.2 for Mac [45, 46]. The data set was further structured as follows: 211 subjects × 12 choice tasks per subject (2,532 choice sets) × 2 alternatives per choice set (5,064 alternatives). Alternatives were assigned 0 if the respondent did not choose this alternative and 1 if he or she chose the alternative in the choice set.

Effects coding was used (−1, 0, 1) for all seven attributes associated with two or three levels of the attributes within the model calculation. The worst level (i.e., highest probability of a negative impact on the decision) in each case was determined as the reference category and assigned −1.

All GLLAMM models were fitted using a multi-nominal logit link function and binomial distribution for the binary response. First of all, a main effects model (one-class model) that assumes that the population of respondents is homogeneous in the weighting (expected utility) was calculated for each attribute. In addition, “multi-group models” (2 class, 3 class, and 4 class GLLAMM models) were calculated, by specifying latent class models using a discrete random coefficients vector. Identification of groups made it possible to identify patterns related to these groups using structural variables. The group models thus provided information about the likelihood of possible subjects falling into a specific group on the basis of the respective structural variables. The model estimates in terms of structural parameters within the latent class model were subsequently calculated, which reflects the strength of influence of the individual structure parameters in each class. “Likelihood ratio tests”, Akaike information criterion (AIC), and Bayesian information criterion (BIC) were used to check the accuracy of the model, to determine the most appropriate model and to test for parameters that might improve the model fit.

Results are reported as estimated parameters of the main-effects model (1 class) and of a latent-class model with three classes. While we did not include a cost attribute, preference estimates were rescaled into progression-free survival equivalents analogous to willingness to pay (WTP) using a nonmonetary numeraire. For interpretation of “progression-free survival” as “currency,” it was possible to evaluate the marginal rate of substitution in relation to the other risk attributes. The information can be derived via how much time a respondent was willing to “give up” for an improvement from a severe side effect to a mild one.. Or vice versa, how much of a side effect a respondent was willing to accept to get one more (progression**-**free) month.

## Results

### Respondent characteristics: socio-demographic data

In the total sample 64.5 % of participants were male and the mean age was 58.9 years (min 29, max 82, SD 8.44). 68.2 % of the subjects stated that they were married. Participant education followed a normal distribution with 52.1 % cumulative disclosures of an intermediate high-school diploma or technical college qualification.

40.3 % of respondents were retired with 32.7 % currently incapable of working. 11.8 % of the respondents were still in part- or full-time jobs. 6.6 % of the respondent had received their NSCLC diagnosis less than 6 months ago, 13.8 % 6 months to 1 year ago, 36.0 % 1–2 years ago and 34.1 % of people had been diagnosed 2–5 years ago. 9.0 % reported that they had been diagnosed more than 5 years ago.

14.7 % of respondents said that they rated their current health as “good” or “very good”. 52.6 % rated their health as “satisfactory” and 32.7 % indicated that their current health was “less good” or “poor” (Table 1).

**Table 1:** Socio-demographic structure of patient sample (*N* = 211)

Sample characteristics (*N* = 211)	Percentage (%)	*N*
Gender		
Male	64.5	136
Female	35.5	75
Age groups (years)		
54 years and younger	26.1	55
55–64 years	46.0	97
65 years and older	28.0	59
Mean		58.9^a^
Marital Status		
Married	68.2	144
Widowed	9.0	19
Divorced or separated	10.4	22
Single	4.3	9
In a relationship, but not married	8.1	17
Education level		
No degree	2.4	5
Junior/middle school certificate	16.1	34
Intermediate high school certificate, secondary school certificate	27.0	57
Vocational school/advanced technical certificate	25.1	53
Abitur, high school diploma, in Germany: university entrance qualification	14.1	31
Technical college degree	7.6	16
University degree or higher	7.1	15
Employment status		
Employed full-time	3.3	7
Employed part-time	8.5	18
Self-employed	1.9	4
Homemaker/housewife	11.8	25
Retired	40.3	85
Disabled/unable to work	32.7	69
Unemployed	1.4	3
Time of NSCLC diagnosis		
Less than 6 months ago	6.6	14
6 Months–1 year ago	13.7	29
1–2 years ago	36.0	29
2–5 years ago	34.1	72
More than 5 years ago	9.0	19
Not sure	0.5	1
Treatment status-current treatment^a^		
No treatment, but regular monitoring/check-ups	33.2	70
Surgery	0.9	2
Radiotherapy	20.4	43
Drug therapy (including chemotherapy)	82.5	174
Other	0.5	1
Treatment status-previous treatment^a^		
No treatment, but regular monitoring/check-ups	8.1	17
Surgery	64.9	137
Radiotherapy	77.3	163
Drug therapy (including chemotherapy)	84.8	179
Other	–	–
With whom do you live in a household?^a^		
Spouses	77.3	163
One or more adult children (18 years or older)	13.7	29
One or more minor children (under 18 years)	8.5	18
Parents/parent	–	–
I live alone	19.9	42
Others	1.4	3
Within your treatment how severe did the side effect diarrhea occur?		
Not at all	12.3	26
Mild	52.1	110
Moderate	29.4	62
Severe	5.2	11
Not sure	0.9	2
Within your treatment how severe did the side effect fatique occur?		
Not at all	8.5	18
Mild	26.1	55
Moderate	37.4	79
Severe	19.4	41
Not sure	8.5	18

^a^Multiple replies possible

### Results of the main effects model

A total of *N* = 211 complete records were transferred to the database and included in the final quantitative data analysis.

The basic model displayed a significant coefficient for all attributes included. This shows that, independent of the placement, all attributes were relevant to the patients’ decision (Table 2). It can be deduced therefore that all attributes included in the decision model have an impact on the therapeutic decisions made by NSCLC patients.

**Table 2:** Estimated parameters of the main-effects model (1 class)

Attribute	Coeff.	Odds ratio	SE	95 % confidence interval		*P* value	*z*
				UB^a^	LB^b^		
Progression-free survival	1.087	2.966	0.0525	1.1902	0.9846	0.000	20.73
Rash	0.432	1.540	0.0396	0.5094	0.3541	0.000	10.89
Nausea and vomiting	0.605	1.832	0.0481	0.6995	0.5109	0.000	12.58
Diarrhea	0.427	1.533	0.0396	0.5051	0.3498	0.000	10.79
Fatigue (tiredness)	0.423	1.527	0.0380	0.4977	0.3486	0.000	11.13
Tumor-associated symptoms	1.090	2.975	0.0562	1.2005	0.9801	0.000	19.39
Mode of administration	0.141	1.152	0.0311	0.2023	0.0804	0.000	4.55

^a^Upper bound

^b^Lower bound, number of level 1 units = 5,064, log likelihood = −980.06933

The main effects model (linear model), which assumed a homogeneous distribution within the population of participants, showed that “progression-free survival” (Coef.: 1.087; OR.: 2.966) and “tumor-associated symptoms” (cough, shortness of breath, and pain) (Coef.: 1.090; OR.: 2.975) are in first place in the preference weights (no significant difference). Because of the coding used in the statistical model, the highest coefficient shows the highest influence in the decision model. Specifically, for an attribute to improve from the worst to the best level (manifestation), the attribute with the highest coefficient will also act as the strongest stimulus to the decision.

The third position is occupied by “nausea and vomiting” (Coef.: 0.605; OR.: 1.832), followed by “rash” (Coef. 0.432; OR 1.540), “diarrhea” (Coef. 0.427; OR 1.533) and “tiredness and fatigue” (Coef. 0.423; OR 1.527); these attributes are also very close to each other. The group of side effects can be considered together to facilitate later interpretation.

The “mode of administration” was estimated to be in the last position (Coef.: 0.141; OR.: 1.152). It exerts by far the least impact on the therapy decision in comparison to other patient-relevant attributes.

### Results of the latent class model and analysis of subgroups

#### Preference differences depending on structural variables

To identify possible subgroup differences starting from the initial main effects model a two-class model and, subsequently, a three-class model were generated. Based on examination of the model fit using AIC and BIC (both indicators for the assessment of overall quality of models) the three-class model could be identified as suitable for illustration of existing heterogeneity in response behavior as well as for representing the probable allocation of subjects per group. As the choice-tasks contained systematic overlap of the middle level based on the experimental design only the coefficients for the first and third level could be evaluated. Therefore the linearity assumption between the levels has to be applied within all estimations.

Seven structural or personal variables were identified to explain the impact on a subjects’ probability of class membership. Significant indicators for group differences were “previous treatment,” “education: vocational school,” “one or more adult children (18 years or older),” “currently undergoing treatment with radiotherapy,” “marital status: widowed and/or divorced,” “high burden of side effects: diarrhea” and “not sure” for the side effect “fatigue.” The three different preference patterns (represented by the relevant classes) (see Fig. 3) were less dependent on the socio-demographic factors included in the questionnaire.

The socio-demographic characteristics “age” and “gender” did not influence the calculated latent class model. It is concluded that these characteristics have no influence on preference in the present decision-making model.

#### Preference patterns class 1

The preference pattern of class 1 [*N* = 47 (22.6 %)] is similar to the linear model for estimation of the main effects (relative importance). The attributes “progression-free survival” and “tumor-associated symptoms” are weighted the highest. The attributes “diarrhea” and “mode of administration” received a negative coefficient in this model. This suggests that respondents are “willing to accept” these attributes in the selection decision in this class. For the attribute “tiredness and fatigue” no significant coefficient was calculated, which would appear to indicate that this attribute had no effect on the decision of this subpopulation.

#### Evaluation of structural variables for class 1

Class 1 includes a below-average number of people with adult children in the household (as opposed to class 3). Higher preference for infusion (indicated by the negative sign) might be due to the fact that persons in this class might prefer to be treated at a hospital and to have direct access to medical care if needed (as this group has below-average adult children within the household). An above-average number of patients with a vocational education can also be found in class 1. The patients matched in class 1 have all undergone previous treatment and none of the patients indicated that they ever received “no treatment and only regular checks.” “No treatment” periods refer to the interval between two chemotherapies, for example, or when a decision has been made to stop treatment and to control the progression of the tumor only. Since this is a deterministic context this cannot be represented in the model. Moreover, the significance of this parameter is rather low.

#### Preference patterns class 2

The preference pattern of class 2 [*N* = 85 (40.5 %)] is comparable with that of class 1 and with the main effects model in respect to the importance of the attributes “progression-free survival” and “tumor-associated symptoms.” One characteristic of the second class, however, is that the side effect attributes have increased substantially in importance and the differences between the outcome attributes and the side effect attributes are lower than in either the first group or in comparison with the main effects model.

#### Evaluation of structural variables for class 2

Class 2 has fewer participants currently in radiotherapy. On the other hand, there are an above-average number of patients who state that they are widowed. Also, relatively fewer people are divorced. Furthermore, persons with the class 2 preference patterns had experienced on average an increased burden from the side effect “diarrhea” in their therapy. Identical to class 1, class 2 only includes patient with previous treatment. None of the patients indicated that they received “no treatment and only periodic checks.”

#### Preference patterns class 3

It is striking that the preference pattern in class 3 [*N* = 79 (36.9%)] is relatively the same across all seven attributes. Furthermore, the attributes “progression-free survival” and “tumor-associated symptoms” are not ranked the highest in class 3, but the difference in the preference weights is rather low.

#### Evaluation of structural variables for class 3

The people in class 3 were more likely to live with adult children. In contrast to class 2 an above-average number of people in class 3 stated they were currently having radiotherapy. Also in contrast to class 2, people indicated that they had experienced fewer episodes of “diarrhea” during therapy. Persons who have experience with control sequences in therapy and periods of “no treatment” are predominantly found in class 3. And finally, all patients in class 3 have indicated “not sure (if adverse event was experienced)” in response to the question about fatigue and tiredness experienced as a side effect.

#### Verification of model performance using a likelihood ratio test

In addition to representation of the proportional distribution within the latent class regression model a likelihood ratio test was performed. It is possible to determine, based on the results of the likelihood ratio test for different quantity of classes, which model parameters improve the quality of the three-class model. The likelihood ratio test also takes into account the increase in the degrees of freedom which can result in the regression model yielding divergent results. It should also be noted that in this calculation all covariates were included directly in the model and thus estimates of the size of classes can be affected as can the parameter estimates of the attributes. In other words, by using the likelihood ratio test it was determined how far the model fit can be improved if the estimated coefficients are set dependent on a covariate (based on the estimated coefficients and related SE displayed in Table 3). In total three different models were estimated and compared; the three-class model performed best.

**Table 3:** Estimated parameters of latent-class model

	Parameter (standard error)		
Attribute	Class 1 coef. (SE)	Class 2 coef. (SE)	Class 3 coef. (SE)
Progression-free survival	4.032 (0.9151)***	3.608 (0.8595)***	0.611 (0.0805)***
Rash	0.436 (0.1345)***	0.737 (0.1967)***	0.775 (0.0840)***
Nausea and vomiting	0.883 (0.1995)***	1.603 (0.4336)***	0.854 (0.0866)***
Diarrhea	−0.781 (0.1776)***	1.585 (0.3491)***	0.668 (0.0701)***
Fatigue (tiredness)	0.034 (0.3603)*	1.305 (0.3144)***	0.514 (0.0709)***
Tumor-associated symptoms	2.442 (0.4874)***	3.574 (0.6994)***	0.726 (0.0890)***
Mode of administration	−1.378 (0.5593)**	0.912 (0.2594)***	0.142 (0.0556)^**^
Proportion of patients per class	47 (22.6%)	85 (40.5%)	79 (36.9%)
Constant per class	−0.4895 (0.2075)	0.0915 (0.2208)	–
Class-membership parameters			
No treatment, only checkups (previous treatment)	–	–	++
Education: vocational school	++		
Household: one or more adult children (18 years or older)	–		++
Currently in treatment with radiotherapy		–	++^a^
Marital status: widowed and/or divorced		++	
High burden of side effects: diarrhea		++	
”Not sure” with side effect “fatigue”			++

*** p < 0.0001, ** *p* < 0.001, * p = not sign, − below average number of patients, + above average number of patients

ª This includes only patients who were treated

The parameters in the model specify whether the probability of class membership is increased or decreased by a structural feature. If a parameter is positive and significant this increases the probability of class membership. For example, if a parameter for class 2 is positive and significant this means that a person with this characteristic is more likely to belong to class 2 than class 3. In reviewing the model the variables “currently in treatment with radiotherapy,” “currently in treatment with medication” (including chemotherapy), “side effect: fatigue” (in all four levels) and “household type: one or more adult children (18 years or older)” were identified as predictors for the improvement of the model quality. The exploratory nature of this approach should be noted and the fact that in multiple testing of all structural parameters the risk of random statistical significance cannot be excluded. This analysis can thus be understood as a complement to the latent class model. The probability of class membership in class 1 or 2 is positively determined by the properties “current treatment: medical treatment (including chemotherapy)." These were the classes in which “progression-free survival” and “tumor-associated symptoms” were considered to be the most relevant. Similarly, there is an increased probability of the class including people who experienced the “side effect: fatigue.” Patients who disclosed “side effect fatigue: not sure” were more likely to pertain to class 3 (see also regression model). An increased class membership for class three is triggered by the properties “current treatment in radiotherapy” and “household type: one or more adult children (18 years or older).” This is in direct concordance to the regression model and is thus confirmed.

## Discussion:

### Interpretation of DCE results

#### Descriptive results

Distribution of the present sample is similar to the systematic review of Blinman and colleagues [23], which exclusively determined preference studies on NSCLC chemotherapy. In concordance, the majority of those affected were male (65%) and the average age was about 58 years and thus in the second half of life. The current treatment guidelines mention a median age of 67 years for German NSCLC patients in stage III and IV while the present sample is somewhat younger [10].

Consistent with the literature, the majority of the included subjects were married, in so far as the data available from other studies allowed for this comparison. The present study includes by far the highest number of NSCLC cancer patients. A total of *N* = 211 NSCLC patients were included in the present study while comparable studies included a range of 56 to 100 cancer patients (not all NSCLC) [23, 47]. Owing to the severity of the indication this constitutes a unique characteristic of the study.

#### Relative importance

Similar to results obtained in the US context [47] the attributes “progression-free survival” and “tumor-associated symptoms” were identified as the two key patient-relevant characteristics in the present study. This implies that the sole consideration of “progression-free survival” as the foundation for decisions is not sufficient from the patients' perspective and multiple criteria are important. Patient benefit is determined by "progression-free survival" and "tumor-associated symptoms" in approximately equal measure. Therefore, both features should be considered in allocation and treatment decisions. In the present decision model the patient benefits are determined by "progression-free survival" and tumor-associated symptoms as well as side effects. Therefore multi-criteria decision models should be taken into account in allocation and treatment decisions.

 In contrast to a study from the US, which ranks “fatigue and tiredness” in first place of all included side effects [47], the patients in the present study assessed the side effect “nausea and vomiting” as the most important side effect in their treatment decisions.

A study by Dubey et al. demonstrated that 73 % of NSCLC patients report they make treatment decisions on the basis of potential side effects if an approximately equal effectiveness is expected [27]. In the present study and in line with Dubey et al. the side effects were equally rated as the second most important block in addition to the effectiveness of treatment and tumor-associated symptoms. Therefore, side effects have a significant impact on the treatment decision. Following the main outcome attributes the possible side effects were weighted by the patients. “Nausea and vomiting” was rated higher than “rash.” A possible explanation might be that sufferers are severely affected by “nausea and vomiting” in their daily life. The patients associate nausea and vomiting with a restriction of their activities of daily living and possibly with a reduction in their quality of life. A “rash” tends to be connected with the need for medical intervention; “Rash” is treated and corrected and thus might be perceived as “less restrictive."

The expectation of benefit from all attributes included show that a high acceptance by patients can be expected for the use of a drug therapy that provides these properties [4]. The inclusion of patient preferences in therapeutic decisions implies stronger patient focus and can be simultaneously valuable for the development of drug therapy in the indication of NSCLC.

#### Subgroup effects

Different studies investigated which patient characteristics (structural variables) could have an influence on the formation of preferences and obtained differing results. One study showed a dependence on educational status, whereas another study found no correlation. Also, different relations were found regarding the age variable. Only the variable “sex” consistently showed no correlation to preferential results in all studies. This is also in accordance with the present results [24, 25, 49–51].

Individual effects of the subgroup analysis applied in this study are in concordance with the results of Blinman et al. [23]. Similarly, no relevant differences in preference patterns by socio-demographic characteristics such as age and sex could be determined.

#### Marginal rate of substitution and time equivalent

Only a few studies have explored deriving clinically-relevant time-equivalents from DCE data. Following standard consumer theory the marginal rate of substitution (MRS) between attributes can be obtained by calculating the ratio of the partial derivatives of each attribute. This calculation addresses the question of how many side effects respondents were willing to accept in order to get one more progression-free month. For one additional month without tumor progression respondents were willing to accept:

- An increase of 0.359 from mild to severe side effects of skin (rash).
- An increase of 0.257 from mild to severe nausea and vomiting.
- An increase of 0.364 from mild to severe diarrhea.
- An increase of 0.367 from mild to severe tiredness/fatigue.
- An increase of 0.142 from mild to severe tumor related symptoms.

In the presence of a linear additive (indirect) utility function compensating estimates can be calculated for a marginal change from mild to severe for each risk attribute by dividing the coefficient of the risk attribute by the coefficient of the time attribute (time without tumor progression). The time equivalent (TE) is the mean maximum time a respondent was willing to forego for an improvement in a single risk attribute. For an improvement from severe to mild in each risk attribute respondents were willing to forego:

- 2.782 months for an improvement in side effects of skin (rash).
- 3.896 months for an improvement in nausea and vomiting.
- 2.750 months for an improvement in diarrhea.
- 2.724 months for an improvement in tiredness/fatigue.
- 7.019 months for an improvement in tumor related symptoms.

#### Assignment of different models

To be able to derive implications for the usage within, for example, an HTA context, the linear model would be needed in the first place because it presents a weight for each attribute. Analysis and explanations of the heterogeneity within the latent class model could help shared decision making by visualising different patient clusters and different need structures. Clustering of patients indicated within the latent class might help the physician to identify patient preference and use a patient-centered treatment. Furthermore, within an HTA context the latent class model could help to support the approval of a treatment option within specific subgroups.

### Limitations of the study

Firstly, recruitment was conducted via a market research company. This could have influenced the study population with respect to individual parameters that could not be examined in total. Access to the market research company is, however, free to everybody. Analysis of the socio-demographic data showed a pattern similar to other studies.

New methodological discussions address the issue of potential rescaling effects within preference estimates. This would relate to the attribute progression-free survival equivalents analogous to WTP using a non-monetary numeraire. During the pretest participants were fully aware of the progression-free survival time and the fact that it differed between levels. We estimated the MRS and a TE. Given the importance of obtaining a valid estimate of the marginal utility of time, a validity test of sensitivity to scope should have been included to test the hypothesis that respondents paid attention to absolute time levels and did not interpret the time levels simply as “low,” “medium” and “high.” As this was not part of the experimental design any discussion of this issue can only be addressed as a demonstration of the need for further research.

It should be mentioned that the patients' evaluation with respect to "rash" (even if it does not play a dominant part in weighting) might change given the fact that an increased rash may be an indicator of a positive reaction to therapy, as demonstrated in several studies [48]. Since “rash” in the present study should be included solely as a side effect this observation was not relevant.

As other studies have also shown, preferences can depend on the cultural background of the study samples and the existing healthcare system as a context factor [23]. When interpreting the study results it is important to remember that a German study sample was used. Furthermore, it cannot be ignored that interaction might be present. Since the experiment was not designed to discern interactions this would need to be addressed in a future experiment.

It is further noted that study participants did not make decisions with their relatives or their physicians, which could differ depending on the patients and their healthcare provider setting. Different stakeholders may have different preferences. As part of an evaluation process it should be possible to take any perspective into account, i.e., that of decision makers, ordinary citizens, patients, the insured or experts. Information about individual views and priorities is necessary.

## Conclusion

Considering the current buzzwords such as “patient involvement” and “shared decision-making” patient preferences should be understood and analysed and the objectives of NSCLC patients regarding therapies and medications should be known.

NSCLC treatment decisions are very complex and require a fine balance between potential outcomes and simultaneous potential risks and side effects. Present data show patients give highest weighting to “progression-free survival” and “tumor-associated symptoms.” Thus, reduction of “tumor-associated symptoms” and the increase in “progression-free survival” are of crucial importance for NSCLC patients in the context of possible therapies. Potential side effects and the associated impairments are traded against them.

This study showed DCEs are appropriate for identifying and weighting patient-relevant characteristics of NSCLC treatment options in terms of possible treatment alternatives. The DCE features a high degree of realism and is easy for the patient to handle.

In this study the essential decision criteria for an optimal drug therapy for the treatment of NSCLC from the NSCLC patients’ view were obtained by using a comprehensive theoretical as well as application-oriented overview of the use of a DCE. Also, patient-relevant outcome measures and the weight attached to different treatment properties and their characteristics by NSCLC patients were determined. Thus, this study revealed how patients evaluate different aspects of drug therapy. These data therefore can be used to add patient evidence to clinical evidence when making treatment or HTA decisions and therefore to extend available knowledge. Incorporating patient perspective into treatment and reimbursement decisions can optimize allocation of scarce resources. In the context of multi-criteria decision-making, a cardinal scale ranking weighting coefficients can be used for the aggregation of patient benefit to assess overall benefit compared to the comparator drug.

Despite the above-mentioned limitations, the results are of high importance. Thus, the DCE provides a practical approach that can help improve communication between patients and providers. In addition, the method used in DCEs has the potential to support clinical and allocative decision-making and to improve the quality of patient care in the long term. Thus, therapies can be designed, assessed and chosen on the basis of patient-oriented findings. As such, a more effective and efficient care of patients can be achieved and benefits increased [4].
